# Supplementary material for: Current Applications and Future Development of Magnetic Resonance Fingerprinting in Diagnosis, Characterization, and Response Monitoring in Cancer
Source: Cancers (Basel). 2021 Sep 22;13(19):4742. doi: 10.3390/cancers13194742 (PMC8507535; doi:10.3390/cancers13194742)
Supplement: Supplementary file 1 [file cancers-13-04742-s001.zip › cancers-1363658-SI.pdf]

Table S1. overview of published MRF oncological application studies.

Title	First Author	Tumour Type	Specific Method Used	Sample Size	Performance	Limitation
MR fingerprinting of adult brain tumors: initial experience [40]	C. Badve	Adult brain tumours	Patients were scanned at 3T using a 20-channel head coil; 3–5 MRF sections were acquired through each lesion; final output consisted of T1, T2, proton-density, and off-resonance maps; FOV = 300 × 300 mm^2; matrix = 256 × 256; section thickness = 5 mm; acquisition time per slice was 30.8 s.	31 patients	Mean T2 values of solid tumour regions of lower grade gliomas differed from metastases (mean, 172 ± 53 ms and 105 ± 27 ms, respectively; p = 0.004); mean T1 of peritumoral white matter around lower grade gliomas differed from that around glioblastomas (mean, 1066 ± 218 ms and 1578 ± 331 ms, respectively; p = 0.004).	Small sample size in a single centre with various tumour types
Radiomic analysis of magnetic resonance fingerprinting in adult brain tumors [41]	S. Dastmalchian	Adult brain tumours	MRF acquisition was performed as in C. Badve's study. Using regions of interest on T1 and T2 maps, second-order texture features were calculated from grey-level co-occurrence matrices and grey-level run length matrices. Selected features were compared across different tumour groups.	31 patients	Low-grade gliomas and glioblastomas had significantly higher run percentage, run entropy, and information measure of correlation 1 on T1 than metastases ($p < 0.017$). The best separation of all three tumour types was seen utilizing inverse difference normalized and homogeneity values for peritumoral white matter in both T1 and T2 maps ($p < 0.017$).	Small sample size in a single centre with various tumour type; single slice two-dimensional acquisition of MRF data did not provide whole tumour coverage
Magnetic resonance fingerprinting to characterize childhood and young adult brain tumors [42]	P. de Blank	Primary low-grade glioma and high-grade tumours	Patients were scanned at 3T using a 20-channel head coil; T1 and T2 values were recorded in regions of solid tumour (ST), peritumoral white matter (PWM), and contralateral white matter (CWM); FOV = 300 × 300 mm^2; in-plane resolution = 1.17 × 1.17 mm^2; slice thickness = 5 mm.	23 patients (14 children and 9 young adults)	Median scan time for MRF and a sequence for tumour localization was 11 min. MRF-derived T1 and T2 values distinguished ST from CWM (T1: 1,444 ± 254 ms vs. 938 ± 96 ms, p = 0.0002; T2: 61 ± 22 ms vs. 38 ± 9 ms, p = 0.0003) and separated high-grade tumours from low-grade tumours (T1: 1,863 ± 70 ms vs. 1,355 ± 187 ms, p = 0.007; T2: 90 ± 13 ms vs. 56 ± 19 ms, p = 0.013). PWM was distinct from CWM (T1: 1,261 ± 359 ms vs.	Sample size; not all tumours were biopsied to prove their classification

					933 ± 104 ms, $p = 0.0008$; T2: 65 ± 51 ms vs. 38 ± 8 ms, $p = 0.008$), as well as from tumour (T1: 1,261 ± 371 ms vs. 1,462 ± 248 ms, $p = 0.047$).	
Application of magnetic resonance fingerprinting to differentiate grade I transitional and fibrous meningiomas from meningothelial meningiomas [43]	R. Zhang	Grade I transitional and fibrous meningiomas and meningothelial meningiomas	All patients underwent conventional MRI, MRF, and DWI scans using a 3 T scanner with a 20-channel head coil to investigate the difference in quantitative T1 and T2 values derived from MRF and apparent diffusion coefficient (ADC) values between different tumour groups; field of view = 256 × 256 mm^2; matrix = 256 × 256; slice thickness = 5 mm.	46 patients	The T1 values (mean ± standard deviation) for meningothelial, transitional, and fibrous meningiomas were 1,661 ± 222, 1,491 ± 91, and 1,485 ± 78 ms, respectively. The T2 values for meningothelial, transitional, and fibrous meningiomas were 87 ± 40, 69 ± 15, and 65 ± 9 ms, respectively. The T1 and T2 values yielded from MRF were able to differentiate WHO grade I transitional and fibrous meningiomas from meningothelial meningiomas.	Sample size; exclusion of other meningioma subtypes; a lack of grading studies
Initial assessment of 3D magnetic resonance fingerprinting (MRF) towards quantitative brain imaging for radiation therapy [49]	L. Lu	Primary brain tumours	A fast whole-brain 3D-MRF sequence initially developed for diagnostic radiology was optimized using flexible body coils for radiotherapy treatment planning and for MRI guided treatment delivery.	3 healthy volunteers	The optimized sequence acquired whole-brain T1, T2, and proton density maps with a resolution of 1.2 × 1.2 × 3 mm^3 in 10 min. The intra- and inter-scanner variability of the intensity-normalized MRF T1 was 1.0% ± 0.7% and 2.3% ± 1.0% respectively, in contrast to 5.3% ± 3.8% and 3.2% ± 1.6% from the normalized T1-weighted MRI.	Sample size; the inter-scanner variability was only evaluated with two 3T scanners from the same vendor
Non-invasive tumor decoding and phenotyping of cerebral gliomas utilizing multiparametric ^{18}F-FET PET-MRI and MR fingerprinting [57]	J. Haubold	Primary brain tumour	PET-MR imaging was performed on an integrated 3 T PET-MR system; PET was acquired in one bed position with an acquisition time of 20 min utilizing a dedicated 16-channel head-and-neck radiofrequency coil;	42 patients	^{18}F-FET PET-MRI and MR fingerprinting enable imaging-based differentiation of low-grade vs. high-grade gliomas and prediction of the mutational status of ATRX, IDH1, and 1p19q. (The 5-fold cross-validated area under the curve for mutations predicting was 85.1% for ATRX, 75.7% for MGMT, 88.7% for IDH1,	Sample size; did not include all potential mutations of gliomas (e.g., IDH2 or TP53)

			MRF resolution = 1.2 × 1.2 × 5 mm³; matrix = 256 × 256.		and 97.8% for 1p19q. The area under the curve for differentiating low-grade vs. high-grade glioma was 85.2%.)	
Development of a combined MR fingerprinting and diffusion examination for prostate cancer [62]	A. Yu	Prostate cancer	T1 and T2 mapping were performed with MR fingerprinting with ADC mapping; field of view = 400 mm²; matrix = 400 × 400; resolution = 1 × 1 mm²; slice thickness = 6 mm.	109 lesions	T1, T2, and ADC from cancer (mean, 1628 ± 344 ms, 73 ± 27 ms, and (0.773 ± 0.331) × 10^{-3} mm²/sec, respectively) were significantly lower than those from normal-appearing peripheral zone (mean, 2247 ± 450 ms, 169 ± 61 ms, and (1.711 ± 0.269) × 10^{-3} mm²/sec) (*p* < 0.0001 for each) and together produced the best separation between these groups.	Resolution only high enough for lesion characterization and not for detection; targeted validation was performed with cognitive guidance rather than US–MR imaging fusion or direct in-gantry biopsy
Targeted biopsy validation of peripheral zone prostate cancer characterization with magnetic resonance fingerprinting and diffusion mapping [63]	A. Panda	Peripheral zone prostate cancer	T1 and T2 mapping were performed with MR fingerprinting with ADC mapping; field of view = 400 × 400 mm; matrix = 400 × 400; resolution = 1 × 1 mm²; slice thickness = 5 mm.	104 lesions	Prostate cancer T1, T2, and ADC (mean ± SD, 1660 ± 270 ms, 56 ± 20 ms, and 0.70 × 10^{-3} ± 0.24 × 10^{-3} mm²/s, respectively) were significantly lower than those for prostatitis (mean ± SD, 1730 ± 350 ms 77 ± 36 ms, 1.00 × 10^{-3} ± 0.30 × 10^{-3} mm²/s) and negative biopsies (mean ± SD, 1810 ± 250 ms, 71 ± 37 ms, and 1.00 × 10^{-3} ± 0.33 × 10^{-3} mm²/s).	Resolution only high enough for lesion characterization and not for detection; only peripheral zone lesions were analysed in this study; targeted biopsy correlation was used instead of whole-mount prostatectomy specimens
MR fingerprinting and ADC mapping for characterization of lesions in the transition zone of the prostate gland [64]	A. Panda	Transition zone prostate cancer	T1 and T2 mapping was performed with MR fingerprinting with ADC mapping; field of view = 400 × 400 mm; matrix = 400 × 400; resolution = 1 × 1 mm²; slice thickness = 5 mm.	75 lesions	The T1, T2, and ADC of normal transition zone (1800 ± 150 ms, 65 ± 22 ms, and (1.13 ± 0.19) × 10^{-3} mm²/sec, respectively) were higher than those in cancers (1450 ± 110 ms, 36 ± 11 ms and (0.57 ± 0.13) × 10^{-3} mm²/sec; *p* < 0.001 for all).	Retrospective analysis from a single centre; the section thicknesses for T2-weighted/ADC and MR fingerprinting maps were different

Feasibility of novel three-dimensional magnetic resonance fingerprinting of the prostate gland: phantom and clinical studies [65]	D. Han	Prostate cancer	The mean T1 and T2 values were compared in the peripheral zone, transition zone, and focal lesions; MRF performed had field of view = 160 × 160 mm, resolution = 0.6 × 0.6 mm^2, slice thickness = 3 mm.	90 patients	In the phantom study, the MRF T1 and T2 values showed a perfect correlation with the gold-standard T1 and T2 values ($R > 0.99$). In the clinical study, the T1 and T2 values in the peripheral zone were significantly higher than those in the transitional zone ($p < 0.001$, both). The T1 and T2 values in prostate cancer were significantly lower than those in the peripheral and transitional zones. The higher the grade of cancer, the lower the T2 values.	Sample size; did not divide the prostate cancer lesions according to their location in the peripheral or transition zone
MR fingerprinting for rapid quantitative abdominal imaging [68]	Y. Chen	Metastatic adenocarcinoma in liver	T1 and T2 values were measured by a 3T MRI scanner with field of view = 44 cm × 44 cm; matrix size = 224 mm × 224 mm; resolution = 1.9 mm; section thickness = 5 mm.	20 focal liver lesions	T1 and T2 in metastatic adenocarcinoma were 1673 ± 331 ms and 43 ± 13 ms, respectively, significantly different from surrounding liver parenchyma relaxation times of 840 ± 113 ms and 28 ± 3 ms ($p < 0.0001$ and $p < 0.01$) and those of hepatic parenchyma in healthy volunteers (745 ± 65 ms and 31 ± 6 ms, $p < 0.0001$ and $p = 0.021$, respectively).	Sample size; did not perform three-dimensional acquisition, which was still restricted by imaging speed
Feasibility of quantitative magnetic resonance fingerprinting in ovarian tumors for T1 and T2 mapping in a PET/MR setting [73]	J. Kaggie	Ovarian cancer	Acquired on a standard 3.0T MRI system using a 32-channel abdominal coil. The MRI protocol consisted of standard qualitative clinical sequences followed by a 2D steady-state-free precession (SSFP) MRF sequence. FOV = 380 × 380 mm^2; matrix = 192 × 192; voxel size = 2.0 × 2.0 × 3.0 mm^3; slice thickness = 3.0 mm; acquisition time = 15 s/slice, with 18–22	4 patients	Mean T1 and T2 relaxation times of the borderline tumour were longer by ~20% and ~58%, respectively, compared with untreated HGSOC tumours. The treated patient, with no evidence of remaining tumour, demonstrated lower T1 and T2 values from the other two HGSOC patients by 50–150% for T1 and by 33–50% for T2.	Sample size

	slices per patient, for a total scan time between 4.5 and 5.5 min.	
